# Supplementary material for: Interactive Web-based Annotation of Plant MicroRNAs with iwa-miRNA
Source: Genomics Proteomics Bioinformatics. 2021 Jul 28;20(3):557–67. doi: 10.1016/j.gpb.2021.02.010 (PMC9801042; doi:10.1016/j.gpb.2021.02.010)
Supplement: Supplementary File S1 — Detailed information regarding sRNA-Seq data collection and preprocessing, SVM modeling, qRT-PCR experiment, and syntenic analysis of the wheat genome [file mmc1.docx]

**File S1 Detailed information regarding sRNA-Seq data collection and preprocessing, SVM modeling, qRT-PCR experiment, and syntenic analysis of the wheat genome**

**Collection and preprocess of *Arabidopsis* sRNA-Seq datasets**

A list of 2024 *Arabidopsis* small RNA sequencing (sRNA-Seq) libraries was obtained from *Arabidopsis* Small RNA Database (ASRD, http://ipf.sustech.edu.cn/pub/asrd) [1], and the corresponding raw reads in SRA format were downloaded from Short Read Archive (SRA) database at the National Center for Biotechnology Information (NCBI). After filtering adapters and low-quality reads, clean reads with a length less than 18 nts or more than 26 nts were discarded. For each library, the resulting reads were collapsed into a set of non-redundant reads with a record of the total read depth. Collapsed reads were aligned to the *Arabidopsis* reference genome (TAIR10; ftp://ftp.ensemblgenomes.org/pub/plants/release-47/fasta/arabidopsis_thaliana/dna/Arabidopsis_thaliana.TAIR10.dna.toplevel.fa.gz) using Bowtie aligner v1.2.3 [2] with the parameter *v* = 1 and *m* = 50, allowing for alignments with at most one mismatch and for reads with at most 50 mapping locations. After removing t/r/sn/snoRNA-matched reads (*i.e.*, reads matched to tRNAs, rRNAs, snRNAs, and snoRNAs), 1063 sRNA-Seq datasets for wild-type Columbia ecotype were obtained, each of which had more than one million mapping reads.

**Collection and preprocess of Maize sRNA-Seq datasets**

A total of 195 previously reported sRNA-Seq libraries were used for miRNA annotation in maize [3]. Low-quality reads and adapters were trimmed. The maize reference genome (B73 RefGen_v4; ftp://ftp.ensemblgenomes.org/pub/plants/release-47/fasta/zea_mays) was used for read alignment. After removing t/r/sn/snoRNA-matched reads and discarding reads with a length less than 18 nts or more than 26 nts, clean reads from 195 sRNA-Seq datasets were used for miRNA prediction.

**Collection and preprocess of wheat sRNA-Seq datasets**

To obtain sRNA-Seq datasets in wheat, we queried the NCBI’s SRA database using the following combinations of search terms: "*Triticum aestivum*" [Organism] AND ("ncRNA-seq" OR "miRNA-seq"). This query returned us 399 runs. We further filtered these query results using the criteria: sequenced using the Illumina platform, single-end sequencing, average spot lengths (“AvgSpotLen” column in Table S6) no more than 51, genotype annotated as wild type. Finally, we acquired 95 sRNA-Seq libraries (Table S6). The raw sequencing data for these libraries were trimmed and mapped to the wheat reference genome (IWGSC RefSeq v1.0; ftp://ftp.ensemblgenomes.org/pub/plants/release-43/fasta/triticum_aestivum), using the same parameters as those used for *Arabidopsis*. Reads aligned to t/r/sn/snoRNA sequences were discarded in the downstream analysis.

**High-throughput criteria and machine learning-based approaches**

Detailed criteria for plant miRNA annotation have been described for the application of high-throughput sRNA-seq and the discovery of miRNAs [4]. Specifically, high-throughput criteria include: (1) no secondary stems and large loop in miRNA/miRNA* duplex, and precursor length no more than 300 nts, (2) maximum five mismatches in miRNA/miRNA* duplex and no more than three nucleotides in asymmetric bulges (RNA structure based on RNAfold v2.4.14 [5] and CentroidFold v0.0.16 [6]), (3) include one-nt positional variants (we changed it to three by the definition of miRNA isoforms) of miRNA and miRNA* when calculating precision (abundance bias ≥ 75%), (4) novel miRNAs should meet all criteria in at least two sRNA-seq libraries, and (5) the length of miRNAs ≥ 20 nts and ≤ 24 nts.

To build a machine learning system, miRNA precursors were transformed from nucleotide sequences to numeric values. To perform this transformation, we characterized each miRNA precursor with 218 sequence-based features, 383 structure-based features, and *N* expression-based features (*N* is the number of sRNA-Seq libraries used in miRNA annotation). There are 593 sequence- and structure-based features that were previously used in miRNA prediction [7,8]. Here we include eight additional features, including the length, minimal free energy (MFE), and adjusted minimal free energy (AMFE) of step-loop sequence, the number of sequences in miRNA precursor, abundance bias, strand bias, secondary structure states predicted by RNAfold and CentroidFold. More details regarding these sequence- and structure-based features were available on the webpage of iwa-miRNA project (http://iwa-miRNA.omicstudio.cloud/static/assets/html/index.html).

Using already annotated miRNA precursors as positive samples, iwa-miRNA builds a one-class support vector machine (SVM) classifier to examine whether some miRNA candidates are predicted as novel miRNAs. The one-class SVM is to estimate a function that will take the value +1 in a region where the majority of the data points are concentrated and the value -1 everywhere else. Users can adjust the parameter ν to be a value ranging from 0 and 1, which represents an upper bound on the fraction of outliers in the data. The one-class SVM classifier was built using the ‘svm’ function (type = ‘one-classification’, kernel = ‘radial’, scale = TRUE) of e1071 package implemented in R (http://cran.r-project.org/web/packages/e1071).

**RNA isolation and quantitative RT-PCR**

Total RNA was isolated from a mixed sample of roots, shoots, leaves, and flowers in adult plants of *Arabidopsis thaliana* (ecotype Columbia) using Trizol (Code No. 15596026; Invitrogen) according to the manufacturer’s instructions and was treated with DNaseI (Code No. 2270A; TaKaRa). First-strand cDNA was synthesized using HiScript 1st Strand cDNA Synthesis Kit (Code No. R111; Vazyme). The quantitative real-time polymerase chain reaction (qRT-PCR) experiments were performed with miRNA-specific forward and universal reverse primer (Table S3) using AceQ qPCR SYBR Green Master Mix (Code No. Q121-02; Vazyme) on a CFX96 Real-Time PCR Detection System (Bio-Rad) according to the manufacturer’s instructions. The miRNAs accumulation was normalized with U6 snRNA. Three biological replicates and three technical replicates were performed for each analysis.

**The duplicated miRNAs of wheat homologous groups**

To identify homologous groups of miRNAs in wheat A:B:D subgenomes, we performed synteny analysis based on synteny conservation of homologous gene groups (*i.e.*, homologous genes with the same order and orientation). Firstly, the protein sequences of protein-coding genes were downloaded from the Ensemble Plant database (ftp://ftp.ensemblgenomes.org/pub/plants/release-47/fasta/triticum_aestivum/pep/Triticum_aestivum.IWGSC.pep.all.fa.gz) and then searched against themselves using DIAMOND-blastp v0.9.30.131 [9]. Subsequently, miRNA precursor sequences were used as queries to search against themselves using BLASTN v2.9.0+ [10] with default parameters. Finally, MCScanX v1.0 [11] was used to identify syntenic blocks with at least five collinear genes (or miRNA precursor) pairs with *E*-value < 1E−05. miRNAs within the same syntenic blocks were clustered into homologous groups of different A:B:D configurations (*i.e.*, 1:1:1, 1:1:0, 1:0:1, 0:1:1, 1:0:0, 0:1:0, 0:0:1, and others), according to their relative positions and genomic coordinates.

References

[1] Feng L, Zhang F, Zhang H, Zhao Y, Meyers BC, Zhai J. An online database for exploring over 2,000 *Arabidopsis* small RNA libraries. Plant Physiol 2020;182:685−91.

[2] Langmead B, Trapnell C, Pop M, Salzberg SL. Ultrafast and memory-efficient alignment of short DNA sequences to the human genome. Genome Biol 2009;10:R25.

[3] Xu Y, Zhang T, Li Y, Miao Z. Integrated analysis of large-scale omics data revealed relationship between tissue specificity and evolutionary dynamics of small RNAs in maize (*Zea mays*). Front Genet 2020;11:51.

[4] Axtell MJ, Meyers BC. Revisiting criteria for plant microRNA annotation in the era of big data. Plant Cell 2018;30:272−84.

[5] Gruber AR, Lorenz R, Bernhart SH, Neubock R, Hofacker IL. The Vienna RNA websuite. Nucleic Acids Res 2008;36:W70−4.

[6] Sato K, Hamada M, Asai K, Mituyama T. CENTROIDFOLD: a web server for RNA secondary structure prediction. Nucleic Acids Res 2009;37:W277−80.

[7] Meng J, Liu D, Sun C, Luan Y. Prediction of plant pre-microRNAs and their microRNAs in genome-scale sequences using structure-sequence features and support vector machine. BMC Bioinformatics 2014;15:423.

[8] Cui H, Zhai J, Ma C. miRLocator: machine learning-based prediction of mature microRNAs within plant pre-miRNA sequences. PLoS One 2015;10:e0142753.

[9] Buchfink B, Xie C, Huson DH. Fast and sensitive protein alignment using DIAMOND. Nat Methods 2015;12:59−60.

[10] Camacho C, Coulouris G, Avagyan V, Ma N, Papadopoulos J, Bealer K, et al. BLAST+: architecture and applications. BMC Bioinformatics 2009;10:421.

[11] Wang Y, Tang H, Debarry JD, Tan X, Li J, Wang X, et al. MCScanX: a toolkit for detection and evolutionary analysis of gene synteny and collinearity. Nucleic Acids Res 2012;40:e49.
